# A Genomic Study of Myxomatous Mitral Valve Disease in Cavalier King Charles Spaniels

**DOI:** 10.3390/ani10101895

**Published:** 2020-10-16

**Authors:** Arianna Bionda, Matteo Cortellari, Mara Bagardi, Stefano Frattini, Alessio Negro, Chiara Locatelli, Paola Giuseppina Brambilla, Paola Crepaldi

**Affiliations:** 1 Department of Veterinary Medicine, University of Milan, Via dell’Università 6, 26900 Lodi, Italy; arianna.bionda@studenti.unimi.it (A.B.); matteo.cortellari@unimi.it (M.C.); mara.bagardi@unimi.it (M.B.); stefano.frattini@unimi.it (S.F.); alessio.negro@unimi.it (A.N.); paola.brambilla@unimi.it (P.G.B.); paola.crepaldi@unimi.it (P.C.); 2 Department of Agricultural and Environmental Sciences, University of Milan, Via Celoria 2, 20133 Milan, Italy

**Keywords:** genetics, mitral valve disease, dogs, cavalier King Charles spaniel, cardiology

## Abstract

**Simple Summary:**

Myxomatous mitral valve disease is the most common acquired cardiopathy in dogs. The earliest onset and the highest incidence of the disease is found in cavalier King Charles spaniels. Previous studies have suggested a polygenic inheritance of the disease in this breed. Here, we aim to expand the knowledge of the genetic basis of early-onset myxomatous mitral valve disease in cavalier King Charles spaniels. The selection of cases and controls is a crucial point of the study and is based on clinical, echocardiographic, and genealogical examinations. We perform three complementary genomic analyses that have never been used for investigating this pathology to identify 10 genes differentiating the genomes of the two groups. After examining these genes, we conclude that *HEPACAM2, CDK6,* and *FAH* (which are also related to transforming growth factor β (TGF-β) pathway, probably associated with the development of the disease) are the ones that are most likely involved in the pathogenesis of myxomatous mitral valve disease.

**Abstract:**

Cavalier King Charles spaniels (CKCSs) show the earliest onset and the highest incidence of myxomatous mitral valve disease (MMVD). Previous studies have suggested a polygenic inheritance of the disease in this breed and revealed an association with regions on canine chromosomes 13 and 14. Following clinical and echocardiographic examinations, 33 not-directly-related CKCSs were selected and classified as cases (*n* = 16) if MMVD was present before 5 years of age or as controls (*n* = 17) if no or very mild MMVD was present after 5 years of age. DNA was extracted from whole blood and genotyped with a Canine 230K SNP BeadChip instrument. Cases and controls were compared with three complementary genomic analyses (Wright’s fixation index—F_ST_, cross-population extended haplotype homozygosity—XP-EHH, and runs of homozygosity—ROH) to identify differences in terms of heterozygosity and regions of homozygosity. The top 1% single-nucleotide polymorphisms (SNPs) were selected and mapped, and the genes were thoroughly investigated. Ten consensus genes were found localized on chromosomes 3-11-14-19, partially confirming previous studies. The *HEPACAM2*, *CDK6,* and *FAH* genes, related to the transforming growth factor β (TGF-β) pathway and heart development, also emerged in the ROH analysis. In conclusion, this work expands the knowledge of the genetic basis of MMVD by identifying genes involved in the early onset of MMVD in CKCSs.

## 1. Introduction

Myxomatous mitral valve disease (MMVD) is the most common acquired heart disease in dogs, accounting for approximately 75% of all dogs with heart disease [1], particularly in older dogs and smaller dog breeds [2]. The cavalier King Charles spaniel (CKCS) shows the earliest onset and the highest incidence of MMVD when compared with other breeds [1,3,4,5]. The primary clinical finding in dogs affected by MMVD is a systolic heart murmur, but no murmur might occur in mild cases [6]; thus, an echocardiography is considered as the gold standard for the confirmation and staging of this disease [7].

There is evidence from the literature that, at least in some breeds, such as CKCSs and dachshunds, the hereditary component plays a predominant role in the pathogenesis of MMVD [8,9,10]. This disease, in particular, has been suggested to be inherited as a polygenic trait. In fact, the proportion of offspring with heart murmurs and the intensity of these murmurs are both significantly greater with increased parental severity [8,10]. Moreover, early-onset MMVD, typically found in CKCSs, also appears to be highly heritable. In particular, the heritability is 0.67 ± 0.07 for the degree of the heart murmur and 0.33 ± 0.07 for the presence or absence of the murmur, considering dogs exclusively aged between 4 and 5 years [9].

Previous studies have demonstrated that the linkage disequilibrium in dogs is 10 to 100 times more extensive than in the human genome; therefore, the number of single-nucleotide polymorphism (SNP) markers required for genomic association studies in dogs is considerably lower than in humans [11,12,13,14]. Moreover, the peculiarities of breed structure of dogs (e.g., a small breeding population and the use of popular sires) reduce the within-breed genetic variability, generally making even a small number of cases and controls (from 20 to 100, depending on the type of trait [15]) useful to effectively detect genomic regions that are associated with a particular trait or disease when genotyping just 15,000 SNPs [11,14], which is 10 times less than those contained in the SNP chip used in the present study. 

In the literature, it is possible to find several genomic studies on canine MMVD, as summarized in Table 1. Madsen et al. (2011) identified two *loci* on canine chromosomes (CFA) 13 and 14 that are weakly associated with the development of MMVD via a genome-wide association study (GWAS) of CKCSs [3]. French at al. (2012), in contrast, did not find any evidence for *loci* associated with a mitral valve murmur with a GWAS in this breed, nor regions of highly discrepant homo/heterozygosity. The authors concluded that the familial occurrence of mitral valve murmurs in the CKCS breed is not due to a single major gene effect [16]. Meurs et al. (2017) performed a whole-genome sequencing of 10 CKCSs and 10 dachshunds. They filtered the variants of canine gene orthologs of the human genes known to be associated with MMVD against a database of variants derived from whole-genome sequencing of 98 medium to large dog breeds. The latter were chosen, assuming that the prevalence of MMVD in medium to large dogs is very low, but their phenotypes were not assessed. No variant was found in any of the genes evaluated that were present in at least eight of 10 affected samples, but a single coding variant, predicted to be benign, was found in the *COL5A1* gene in nine of the 10 affected CKCSs and in, at most, 5% of the other breeds examined [17].

The Maltese breed was the object of two genomic studies. Lee et al. (2018) applied a candidate gene polymorphism approach and identified six polymorphisms of the *SERT* gene in samples with MMVD [18]. The GWAS performed by Lee et al. (2019), instead, revealed significant SNPs in several genes associated with cardiac function, including *PDZD2*, *ARVCF*, *CTNNA3,* and *LDLRAD4* [19].

Torres-García et al. (2016) studied the polymorphisms of the *COL1A2* gene, which interestingly localizes on the region of CFA 14 (identified by Madsen et al.) in poodles, and found an association between the rs22372411 variant and susceptibility to MMVD [20].

The problem of all GWAS case-control studies concerning MMVD is the late onset of the disease, making the identification of a real control which will not develop the disease at a later age more difficult. In 2015, Stern et al. tried to solve this problem by associating their results with the whippet dog breed with a continuous variable that included the age of onset of the MMVD (considered early if under 5 years of age) and a score of severity. In this study, a genome-wide significant association was identified on the region of CFA 15, containing *FSTL5,* and of CFA 2, containing *ARHGAP26* [21].

At present, there is no genetic test for MMVD, and the breeding plans for CKCSs put in place so far have not been as effective as expected, especially if based only on auscultatory findings [22,23,24]. Nevertheless, an accurate selection of animals for breeding is essential, since the high prevalence of this pathology in this breed makes the elimination of all the dogs diagnosed with MMVD from reproduction unfeasible. Bagardi et al. [4] clinically and echocardiographically evaluated a representative sample of the Italian population of CKCSs, identifying signs of myxomatous degeneration in 90% of them, also widely present in young dogs. Discovering the genetic basis of MMVD may increase the effectiveness of breeding protocols, allowing an early identification of subjects predisposed to a severe form of this pathology. For this reason, here, we conduct a genomic study on the Italian population analyzed by Bagardi et al. [4]. The selection of cases and controls was a crucial aspect of our study and was based on a complete echocardiographic and genealogic examination, with the latter also being checked against the genomic data. The genomic analyses we performed here have never been used for the investigation of this pathology, and the examination of previously published data about the genes and pathway we identified here also allowed us to describe their possible role in the pathogenesis of the disease. Therefore, the aim of this study was to find genomic regions associated with early-onset MMVD predisposition in the CKCS dog breed. 

## 2. Materials and Methods

This study was carried out with 33 privately owned CKCSs. The main selection criteria were the American College of Veterinary Internal Medicine (ACVIM) classification and age. Our samples were grouped as follows:

- Cases (*n* = 16): Dogs with MMVD (class B1 or more severe) diagnosed before the age of 5 or with severe disease (class C or D) before the age of 8;
- Controls (*n* = 17): Dogs without MMVD (class A) or with extremely mild signs of MMVD (class B1 with a trivial mitral regurgitation characterized by a maximal ratio of the regurgitant jet area signal to left atrium area ≤ 20%) [25] over 5 years of age or those suffering from a mild form of disease (class B1) over 8 years of age.

By way of example, subjects in ACVIM class B2 that were older than 5 years or in more severe classes that were older than 8 years could not be considered as either cases or controls; thus, they were excluded from this study.

Moreover, in order to limit the rate of consanguinity among the dogs as far as possible, in cases of close kinship, the sample with more “extreme” characteristics (the youngest affected or the oldest healthy dog) was chosen.

These dogs were selected among a larger group of 90 CKCSs, examined at the Cardiology Unit of the Department of Veterinary Medicine of University of Milan between December 2018 and September 2019. Informed consent was signed by the owners, in compliance with the ethical committee statement of the University of Milan number 2/2016. 

Information regarding birthdates was verified by checking each animal’s microchip number in the regional registry, while the genealogical study was derived from the consultation of the online genealogy book of the Italian Kennel Club (ENCI) (http://www.enci.it/libro-genealogico/libro-genealogico-on-line#) or the Centrale Canine site (https://www.centra-canine.fr/lofselect).

The cardiovascular system was evaluated by checking the presence/absence of a murmur and, if present, its intensity (grade I–VI/VI) and point of maximum intensity.

Since auscultation in dogs with echocardiographic evidence of this disease has often proven to be normal, in addition to clinical data, all subjects underwent a complete echocardiographic examination that was performed by three well-trained investigators using a MyLab50 Gold cardiovascular ultrasound machine (Esaote, Florence, Italy). The exams were carried out according to a standard procedure with concurrent continuous electrocardiographic monitoring [26]. Dogs were staged according to the ACVIM guidelines [7].

Peripheral venous blood sampling was performed at the end of the examination. Blood was collected from the jugular vein into 2.5 mL EDTA tubes after a 12-h fasting period.

### 2.1. Statistical Analysis

The statistical analysis was performed using JMP^®^ 15.0.0 (SAS Institute Inc., Cary, NC, USA, 1989–2019). Appropriate descriptive analyses were applied. Variables were reported as the mean ± standard deviation if they were normally distributed after an Anderson–Darling test; otherwise, they were reported as median and interquartile range values. Individual and clinical data of the subjects included in different groups were compared with linear regression and χ^2^ tests. The statistical methods applied to the genealogical and genomic analyses are reported below.

### 2.2. Genealogic Analysis

Pedigree information was analyzed using Optisel [27]. The inbreeding coefficient (F) and average relatedness coefficient (AR) were both calculated.

F is the probability of an individual receiving, at one *locus,* two identical-by-descent alleles that are copies of a single allele carried by a common ancestor of the parents. AR is the probability that an allele randomly chosen from the whole population in the pedigree belongs to a given animal.

### 2.3. DNA Extraction and Genomic Analysis

The whole-blood samples, collected in EDTA tubes, were stored at −20 °C. DNA was extracted using a DNeasy Blood and Tissue Kit (QIAGEN^®^, Hilden, Germany) according to the manufacturer’s instructions. The concentration and quality of the DNA of each sample were both assessed using a NanoDrop 1000 spectrophotometer (Thermo Scientific^®^, Waltham, MA, USA). The quality and quantity of the DNA extracted were suitable for the downstream analyses.

All 33 samples were genotyped via outsourcing by Agrotis S.r.l., Laboratory of Genetics and Services, using Canine 230K SNP BeadChips (containing over 230,000 SNPs) on an iScan System (Illumina^®^, San Diego, CA, USA).

Raw genotype data (in the .ped and .map formats) were processed for quality control using the PLINK 1.9 software package [28] as follows: SNPs were excluded if they had a call rate of <95% or if they had a minor allele frequency (MAF) of <1%; SNPs residing on sex chromosomes were excluded because the analysis of males and females alone would have reduced their power; individuals were excluded if the genotyping rate per individual was <95%. The BEAGLE 4.1 software package was used for phasing genotype data [29].

Wright’s fixation index (F_ST_) and single SNP cross-population extended haplotype homozygosity (XP-EHH) were calculated for the SNPs left after the quality control.

F_ST_ is the proportion of genetic diversity due to allele frequency differences between cases and controls groups. Thus, *loci* showing unusually large amounts of differentiation (high F_ST_ values) may identify regions of the genome that have been subject to diversifying selection in the two groups, whereas *loci* showing unusually small amounts of differentiation (low F_ST_ values) may identify regions that have been subject to stabilizing selection [30,31]. F_ST_ was calculated using PLINK 1.9. All markers ranking in the top 1% of the empirical distribution of F_ST_ values were considered as relevant. This threshold, which corresponds to an usual statistical threshold, was used in other similar studies [32,33] to retain only the highest signals.

XP-EHH is a linkage disequilibrium-based method that compares the lengths of haplotypes (consistent with the allele under selection and the neighbor variants in linkage disequilibrium) at each marker between two different populations, allowing the detection of strong, directional selection of one allele in one of the two populations while remaining polymorphic in the other [34,35]. XP-EHH was calculated using the SELSCAN 1.1.0 software package [36]. Similarly to the F_ST_ analysis, all markers within the top 1% of the empirical distribution of normalized XP-EHH values [37,38,39] were considered as relevant. The top 1% SNPs of both the analyses were mapped to the reference genome assembly CanFam3.1.

Since combining multiple independent tests increases power and resolution [35,40], we then compared the results of the aforementioned analyses in order to identify the SNPs found by both of them, namely, the ones that most significantly differentiated between the cases and controls.

The genes containing the relevant SNPs were examined for pathways using Enrichr [41], which looks for associations in various libraries, including KEGG Human 2019, WikiPathways Human 2019 and BioPlanet 2019. Information about the identified genes was obtained via the GeneCards database (https://www.genecards.org/) [42].

Genomic regions subjected to selection may show a reduced nucleotide diversity and increased homozygosity around the selected *locus* if compared to the rest of genome [43]. Therefore, on a subset of genes consisting of consensus genes and genes involved in the most relevant pathways, runs of homozygosity (ROH) were investigated using a sliding window approach in PLINK 1.9. The sliding window was 50 SNPs long, and a maximum of five missing genotypes and no heterozygous SNPs were tolerated. An ROH was called if the following criteria were fulfilled: (1) 50 or more consecutive homozygous SNPs; (2) a minimum length of 1 Mb; (3) a minimum density of one SNP per 50 kb; (4) a maximum gap between two consecutive SNPs of 100 kb. For each group, the proportion of dogs showing a ROH was calculated. The ROH-based inbreeding coefficient (F_ROH_) was calculated for each animal, dividing the total length of all ROH in its genome by the length of the autosomal genome covered by SNPs on the chip.

## 3. Results

Our sample consisted of 33 subjects chosen among 90 CKCSs previously investigated by Bagardi et al. [4]. Thirty-one out of 33 (94%) dogs had signs of mitral valve degeneration. Among the controls, two were classified as A and 15 as B1; fourteen cases were included in ACVIM class B1, one in class B2, and one in class D. This subset was composed of 22 females and 11 males. Age ranged from 0.8 to 11.4 years (6.2 ± 2.6 years). Distributions in the ACVIM classes, age, and gender were not significantly different to the whole sample of 90 dogs. According to the selection criteria, age was significantly higher in controls (8.1 ± 0.5 years) than in cases (4.1 ± 0.5 years) (*r^2^* = 0.53, *p* < 0.0001). 

It was possible to get the pedigree information of 20 out of 33 (61%) dogs (81% of cases and 41% of controls). The average relatedness (AR) and average inbreeding (F) coefficients calculated for the dogs with a pedigree were 0.06 and 0.01, respectively. Since pedigree information was not available for all the dogs included in our study, we also used a genetic marker-based method for calculating inbreeding (F_ROH_). F and F_ROH_ are not directly comparable, because the pedigree-based coefficient measures the mean expected autozygosity of an individual (identity by descent), whereas the latter measures the realized autozygosity (identity by state). F_ROH_ was found to be 0.24 ± 0.03 for the whole sample. No significant differences were found between F_ROH_ in cases (0.24 ± 0.04, ranging from 0.18 to 0.28) and in controls (0.23 ± 0.02, ranging from 0.19 to 0.28). These results demonstrate that the inbreeding of the two groups was similar and, therefore, could not influence the results of our genetic analyses. Moreover, the discrepancy between F and F_ROH_ was consistent with that observed in other studies on dogs [44,45,46].

### 3.1. Genomic Analysis

After the quality control, which excluded the SNPs with a low call rate and MAF and those localized on the sex chromosomes, 103,606 SNPs remained for downstream analyses. None of the 33 animals were excluded.

**Figure 1:**
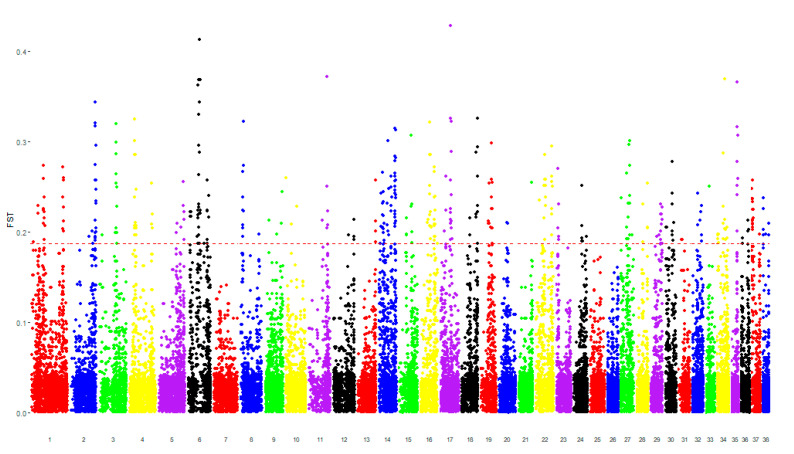
Manhattan plots of the Wright’s fixation index (F_ST_) analysis comparing cases vs. controls. Each single-nucleotide polymorphism (SNP) is represented by a dot. Each chromosome is represented by a different color. The dotted red line represents the cutoff value of the top 1%, equal to 0.19. The genomic regions in which SNPs were mapped over the dotted red line were considered as significantly different between the two compared groups.

**Figure 2:**
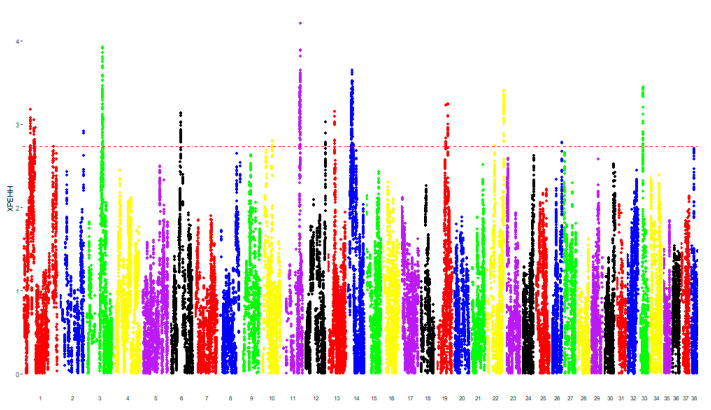
Manhattan plots of the cross-population extended haplotype homozygosity (XP-EHH) analysis comparing cases vs. controls. Manhattan plot of log_10_ XP-EHH values. Each SNP is represented by a dot. Each chromosome is represented by a different color. The dotted red line represents the cutoff value of the top 1%, equal to 2.73. The genomic regions in which SNPs were mapped over the dotted red line were considered as significantly different between the two compared groups.

Comparing the case and control groups, there were 291 SNPs characterized by the top 1% values of F_ST_ (0.19–0.43), mapping regions containing 157 different genes (Figure 1). There were 152 SNPs characterized by the top 1% values of XP-EHH (2.73–3.82), mapping regions containing 45 different genes (Figure 2). The SNPs shared between both the analyses were mapped to genomic regions containing 10 genes (hereafter called “consensus genes” for conciseness), as reported in Table 2. 

**Figure 3:**
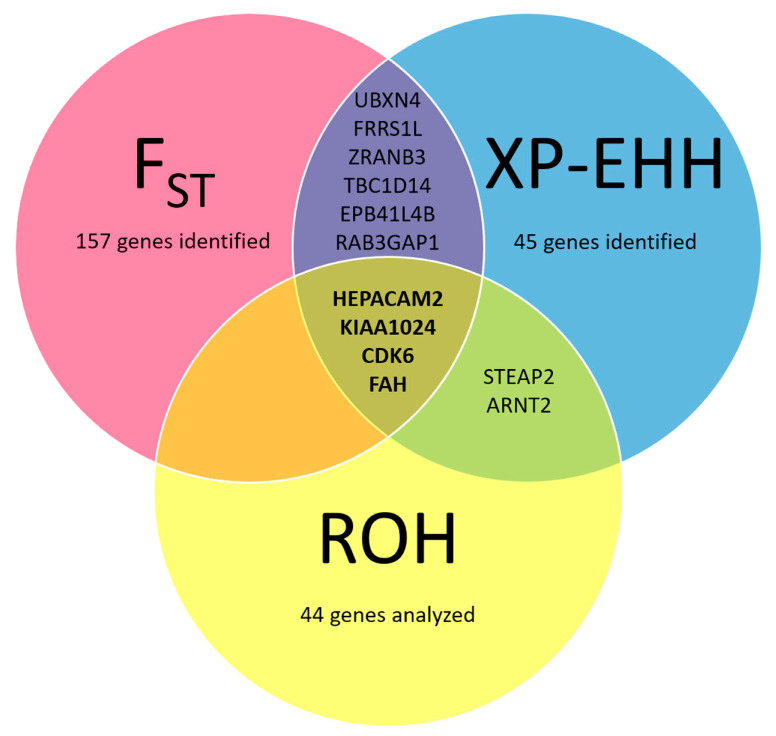
Venn diagram representing the genes identified by the F_ST_, XP-EHH, and runs of homozygosity (ROH) analyses. The SNPs with the top 1% values of F_ST_ and XP-EHH were mapped on genomic regions containing 157 and 45 genes, respectively. The intersection between the two circles represents the consensus genes. For a subset of 44 genes, highlighted by the aforementioned analyses (see Supplementary Materials, Table S1), ROH were investigated: ROH including the *STEAP2*, *HEPACAM2*, and *CDK6* genes were found in at least 50% of cases and no more than 9% of controls; ROH including the *ARNT2*, *KIAA1024*, and *FAH* were present in almost 80% of cases but only in approximately 40% of controls.

ROH analysis revealed that at least 50% of cases and no more than 9% of controls showed homozygous regions around the *STEAP2*, *HEPACAM2,* and *CDK6* genes. The ROH data, including *ARNT2*, *KIAA1024,* and *FAH* were also interesting, because they were present in the genome of almost 80% of cases but only in approximately 40% of controls. No ROH was found in any group for the genes *ADCY9*, *AXIN1*, *CACNA1H*, *CREBBP*, *PDPK1*, *SLC8A2,* and *TRAP1*. All details about the proportion of cases and controls in which ROH were found are shown in the Supplementary Materials (Table S1). Figure 3 represents the most relevant genes identified by our analysis.

### 3.2. Pathway Analysis

To better understand the functions performed by each identified gene, single genes were evaluated in relation to the pathways they are involved in. This allowed classification of 34 of the aforementioned genes in the following most relevant pathways: the Wnt signaling pathway, apelin pathway, hippo signaling pathway, ErbB and epidermal growth factor receptor (EGFR), transforming growth factor β (TGF-β) signaling pathway, endothelins, aldosterone, renin, and body mass index. All details are reported in Table 3.

## 4. Discussion

Among all dog breeds, CKCSs stand out for being the breed with the highest prevalence and the earliest onset of MMVD [1,3]. Therefore, the purpose of this study was to identify selection signatures that are able to distinguish between subjects with a diagnosis of MMVD at a very young age (before 5 years) and subjects in which this pathology may appear at an older age (after 5 years) or otherwise persist at a milder stage for a long time (after 8 years).

Following the calculation of F_ROH_ in cases and controls, we verified that the degree of inbreeding of the two groups was similar. Moreover, our results were superimposable to those found in other studies on various canine breeds [44,45,46]. The identification of consensus genes was reached using two independent methods that analyze different genomic characteristics: F_ST_ highlights genomic differences between groups in terms of expected heterozygosity, whereas XP-EHH is based on the comparison of regions of homozygosity that differentiate the groups. Moreover, ROH analysis on the regions surrounding these genes showed a different presence of long homozygous portions of DNA between the cases and controls. From this analysis, we observed that ROH containing the *HEPACAM2* and *CDK6* genes were present in 50% of cases and only 5% of controls. It could be hypothesized that these genes may be involved in predisposition to rapidly progressing MMVD rather than its early onset. A follow-up of the dogs included in the case group may clarify if the progression of the disease in subjects in which ROH were found around the aforementioned genes is quicker than in subjects in which they were not present. Another possible reason is that the cases in which ROH were absent are heterozygous carriers of the allele that contributes to MMVD predisposition. *KIAA1024* and *FAH* genes were also relevant, because ROH flanking them were found in the genome of 80% of cases but only in approximately 40% of controls. A possible explanation of the presence of controls showing a ROH around these genes is that they might have been misclassified due to the mitral valve pathophysiology, which makes it difficult to detect a real control. 

To better describe their possible role in MMVD onset and progression, an accurate investigation about consensus genes was performed. Some of them were shown to be directly or indirectly related to mechanisms already supposed to be involved in the disease’s pathogenesis, such as the TGF-β signaling pathway, or to processes related to heart development or functionality. The most relevant consensus genes are described below.

*HEPACAM2* interacts with *FGFR1* (fibroblast growth factor receptor 1), which is associated with abnormal heart development. Moreover, during adult life, valves maintain a pool of mesenchymal cells responsive to FGF and producing proteoglycans, which are also increased during MMVD [47]. It should be noted that *HEPACAM2* may localize on the region (CFA 14q1.3) that Madsen et al. (2011) found to be associated to MMVD [3]. 

*FAH* interacts with *ADAMTSL4* (ADAMTS like 4), which is supposed to facilitate *FBN1* (fibrillin 1) microfibril biogenesis [48]. *FBN1* is one of candidate genes for MMVD predisposition, because it regulates TGF-β signaling and is associated with Marfan syndrome, which represents one of the syndromic forms of human mitral valve prolapse [49]. 

*CDK6* prevents cell proliferation and negatively regulates cell differentiation but is required for the proliferation of specific cell types. Moreover, it interacts with *CDKN2B* (cyclin-dependent kinase inhibitor 2B), whose expression was found to be induced by TGF-β and is associated with coronary heart disease [50].

*EPB41L4B* promotes cellular adhesion, migration, and motility in vitro and may play a role in wound healing [51]. This gene interacts with the *CASQ2* (calsequestrin 2) gene, which encodes a protein localized in cardiac muscle cells that stores calcium for muscle function [52]. Mutations in this gene cause catecholaminergic polymorphic ventricular tachycardia [53]. 

It is interesting to note that some genes were associated with height (*CDK6* and *ZRANB3*) or body mass (*FRRS1L*, *EPB41L4B*, and *ZRANB3*) in humans. It has been well established that small dog breeds are predisposed to MMVD [54,55]. Moreover, people affected by mitral valve prolapse tend to have a low body mass index and be leaner and shorter than other individuals [56,57]. A morphometric evaluation of CKCSs could allow identification if the selection for specific physical body features is related to the predisposition to the disease. In fact, the circumference of the thorax has already been negatively correlated with mitral valve prolapse in dachshunds [8]. 

For the other genes able to significantly distinguish between cases and controls, reported in Table 2, no evident correlation with MMVD was found.

The main pathways associated with the genes identified by this study appear to be involved in processes related to heart development and homeostasis, as reported by several studies on humans, mice, and dogs. For example, during valvulogenesis, TGF-β is fundamental for the formation of endocardial cushions and epithelial-to-mesenchymal transition [58,59]. Cell migration and proliferation in these cushions requires the ErbB and Wnt canonical pathway, the latter being also important for maintaining the aforementioned pool of undifferentiated mesenchymal cells responsive to FGF, even in adult life [47,60,61,62]. Valvular interstitial cell (VIC) activation to myofibroblasts, one the most accredited pathogenetic mechanisms of MMVD, seems to be stimulated by TGF-β and inhibited by apelin [63,64]. Canine VIC exposition to TGF-β3, in fact, has been shown to be able to regulate myofibroblast activation and proteoglycan synthesis in an in vitro system [65]. Moreover, the knockdown of ErbB or Axin2 (which represents one of the principal Wnt canonical pathway inhibitors) determined the development of hyperplastic and myxomatous valves in mice in some studies [66,67]. The TGF-β pathway is involved in many human cardiovascular diseases, such as the Marfan, Ehlers–Danlos, Loeys–Dietz, and aneurysms–osteoarthritis syndromes, whose phenotypes often feature mitral valve prolapse [68,69,70]. It is worth mentioning that a study demonstrated that the expression of TGF-β 1 and 3 in cells and the extracellular matrix (ECM) was increased in MMVD-affected canine mitral valves [71]. It has been speculated that the endothelium can be involved in MMVD pathogenesis as well. Its damage upregulates endothelin and nitric oxide, which are involved in the production and alteration of ECMs [72,73]. Moreover, an increase in endothelin receptors has been proven to be associated with MMVD in canine mitral valves [74,75], and with age and mechanical stress in porcine ones [74,75]. Regarding renin and aldosterone, it is well documented that, when the stroke volume decreases due to MMVD progression, several neuro-endocrine compensatory mechanisms are activated, including the renin–angiotensin–aldosterone system (RAAS) [76]. Although this system contributes to the maintenance of blood pressure in heart failure, it is one of the main targets of MMVD therapy due to its role in the development of congestive heart failure itself [77,78]. Finally, the role of platelet activation has not yet been clarified. Human patients with a mitral valve prolapse seem to be more at risk of thromboembolism [79,80,81] and present increased platelet activation [82,83]. In dogs, instead, no significant association has been found between MMVD and thrombus formation [84], and, in most of the studies, platelet functionality has been decreased in canines [85,86,87].

## 5. Conclusions

Several mechanisms have been thought to contribute to the development of MMVD, such as the TGF-β signaling pathway [65,71], increases in serotonin receptors [88,89,90,91], alterations of ECM organization [65,71,89,92], endothelial damage [72,84,89,93,94,95], and oxidative stress [89]. The present genomic study involves a relatively small number of cases and controls. The high prevalence of MMVD in CKCSs, even at a young age, as well as the variability in its progress, makes it difficult to recruit a great number of subjects that can be clearly categorizable as either cases or controls. However, the careful selection of cases and controls among a larger population of dogs made it possible to identify genes and pathways potentially involved in the pathogenesis of early-onset MMVD in the CKCS dog breed. Particularly, our findings are consistent with the hypothesis that the role of TGF-β, ECM disruption, and/or endothelin may be relevant and deserve further investigation.

Genomic studies are not only important to identify genes associated with MMVD predisposition, but also to predict the loss of genetic diversity of the breed following the exclusion of reproduction of dogs with specific phenotypical features. Indeed, a breeding program should not exclude more than 50% of the dog population [96] and, in particular, no more than 30% when screening for a single disease [97]. For example, since the prevalence of MMVD is highly dependent on age, it would be important to choose an age limit whereby dogs with early onset of MMVD can be excluded from reproduction. On the other hand, to place the limit at an advanced age would result in the exclusion of an excessive number of animals for breeding. 

The investigation of the genetic basis of canine MMVD would surely benefit from an increase in the number of samples, which would also permit the analysis of sexual chromosomes (a X-linked form of mitral valve prolapse has, in fact, been found in human [98]). Moreover, a follow-up study conducted on ACVIM B1 patients could allow us to evaluate how MMVD progresses in these dogs. This will be helpful to identify genomic haplotypes associated with early-onset and rapidly progressing MMVD predispositions that, together with morphometric, clinical, and echocardiographic characterizations, could be used as part of a screening program for CKCSs, defining early selection criteria for the exclusion of subjects from breeding. In this respect, it would be important to include collaboration with the veterinary medical community, which may inform us about dogs that should particularly be included in further genomic studies as either a case or control sample.

## Figures and Tables

**Table 1:** Summary of previous genomic studies about myxomatous mitral valve degeneration (MMVD).

Authors	Results	Dog Breed	Sample Size	Criteria for the Inclusion		Diagnostic Techniques	Genomic Analysis
				Age	Diagnosis		
Madsen et al., 2011	CFA 13q2.2.3 CFA 14q1.3	CKCS	139 cases	<4.5 years <8 years	Murmur ≥1/6 and ARJ/LAA ≥20% Heart failure symptoms	Auscultation Echocardiography	GWAS
			102 controls	>8 years	Murmur ≤2/6 and ARJ/LAA ≤50%		
French et al., 2012	No mutations at a single genetic *locus* were found	CKCS	18 early-onset	<5 years	Detectable murmur	Auscultation	Homozygosity mapping GWAS
			18 late-onset	>7 years	Detectable murmur		
Stern et al., 2015	*FSTL5*, *EEF1a1a*, *NAF1*, *NPY1R*, *NPY5R*, *TMA16*, *March1*, *ARHGAP26*	Whippet	138 dogs	5 years cut-off	Scoring based on age, presence and degree of mitral valve prolapse, regurgitation, and left heart enlargement	Auscultation Echocardiography Cumulative echocardiographic score system	GWAS
Torres-García et al., 2016	Allele T of the rs22372411 variant of *COL1A2*	Poodle	50 cases	No restrictions >8 years	Diagnosis of MMVD MMVD absent or mild	Auscultation Echocardiography	Candidate gene polymorphisms
			80 controls	>8 years	MMVD absent or mild		
Meurs et al., 2018	A missense mutation of *COL5A1*, predicted to be benign, was present in CKCS	CKCS and dachshund	10 CKCSs and 10 dachshunds as cases	No restrictions	Diagnosis of MMVD	Auscultation Echocardiography	Candidate gene approach, whole genome sequencing
			98 medium to large dog breeds as controls	No restrictions	Phenotype not evaluated; low prevalence of MMVD		
Lee et al., 2018	*SERT* (*SLC6A4*): c.1193delT (p.Val397Gly)	Maltese	20 cases	No restrictions	Diagnosis of MMVD	Echocardiography	Candidate gene polymorphisms
			10 controls	No restrictions	Echocardiographically healthy		
Lee et al., 2019	*PDZD2*, *CTNNA3*, *LDLRAD4*, *ARVCF*	Maltese	32 cases	No restrictions	Diagnosis of MMVD	Echocardiography Radiography	GWAS
			16 controls	>10 years	Echocardiographically healthy		

CFA: canine chromosome; CKCS: Cavalier King Charles spaniel; ARJ/LAA: area of regurgitant jet/left atrium area ratio; GWAS: genome-wide association study.

**Table 2:** Consensus genes common to the F_ST_ and XP-EHH analyses: names and chromosomal coordinates.

*Gene Name*	CFA	Start	End	Complete Name
*KIAA1024*	3	57739740	57748234	KIAA1024
*TBC1D14*	3	59003766	59097331	TBC1 domain family member 14
*FAH*	3	57300583	57326453	Fumarylacetoacetate hydrolase
*FRRS1L*	11	64281895	64447650	Ferric chelate reductase 1 like
*EPB41L4B*	11	64312862	64447436	Erythrocyte membrane protein band 4.1 like 4B
*CDK6*	14	18188429	18420100	Cyclin dependent kinase 6
*HEPACAM2*	14	18695744	18735539	HEPACAM family member 2
*RAB3GAP1*	19	37861985	37957007	RAB3 GTPase activating protein catalytic subunit 1
*ZRANB3*	19	38002194	38302593	Zinc finger RANBP2-type containing 3
*UBXN4*	19	38519927	38568223	UBX domain protein 4

**Table 3:** Pathways and heart diseases associated by Enrichr to the genes identified by our genomic analyses.

Pathway or Disease	*p*-Value	Adjusted *p*-Value	Associated Group of Genes	Library	Associated Genes
Wnt signaling pathway	0.002	0.040	Top 1% F_ST_	KEGG 2019 human	*CREBBP*, *TCFL1*, *SMAD3*, *AXIN1*, *WNT2*, *PLCB2*
	0.0003	0.031	Top 1% F_ST_ + XP-EHH	Wikipathways 2019, mouse	*CREBBP*, *TCFL1*, *PPP2R2C*, *AXIN1*, *PRKD1*, *WNT2*
Hippo signaling pathway	0.002	0.040	Top 1% F_ST_	KEGG 2019 Human	*LATS1*, *TCF7L1*, *SMAD3*, *AXIN1*, *CTNNA3*, *WNT2*
	0.001	0.028	Top 1% F_ST_ + XP-EHH	KEGG 2019 Human	*LATS1*, *TCFL1*, *SMAD3*, *PPP2R2C*, *AXIN1*, *CTNNA3*, *WNNT2*
Apelin	0.001	0.026	Top 1% F_ST_	KEGG 2019 Human	*ADCY9*, *SMAD3*, *ITPR2*, *ADCY2*, *PLCB2*, *SLC8A2*
	0.002	0.050	Top 1% F_ST_ + XP-EHH	KEGG 2019 Human	*ADCY9*, *SMAD3*, *ITPR2*, *ADCY2*, *PLCB2*, *SLC8A2*
ErbB and EGFR	0.0002	0.047	Top 1% F_ST_	BioPlanet 2019	*ADCY9*, *PDPK1*, *PDE1A*, *ITPR2*, *NRG1*, *ADCY2*
TGF-β	0.009	1.000	Top 1% XP-EHH	BioPlanet 2019	*ARNT2*, *CDK6*, *LPAR1*, *CTNNAL1*, *STEAP2*
Body Mass Index	0.0002	0.313	Consensus	GWAS catalog 2019	*ZRANB3*, *EPB41L4B*, *FRRS1L*, *UBXN4*, *RAB3GAP1*
Endothelins	0.003	0.158	Top 1% F_ST_ + XP-EHH	BioPlanet 2019	*ADCY9*, *ADCY2*, *PLCB2*, *BCAR1*
Aldosterone	0.0001	0.008	Top 1% F_ST_	KEGG 2019 Human	*ADCY9*, *ITPR2*, *ADCY2*, *PRKD1*, *PLCB2*, *CACNA1H*
Renin	0.0002	0.008	Top 1% F_ST_	KEGG 2019 Human	*ADCYAP1R1*, *PDE1A*, *PDE3A*, *ITPR2*, *PLCB2*
Platelet activation	0.003	0.046	Top 1% F_ST_	KEGG 2019 Human	*ADCY9*, *ITPR2*, *ADCY2*, *TLN2*, *PLCB2*

Significant adjusted *p*-values and consensus genes are indicated in bold. EGFR: epidermal growth factor receptor; TGF-β: transforming growth factor β. Adjusted *p*-values were automatically calculated by Enrichr using the Benjamini–Hochberg method for correction for multiple hypotheses testing.

## Supplementary Materials

The following are available online at https://www.mdpi.com/2076-2615/10/10/1895/s1: Table S1. Proportion of cases and controls showing a run of homozygosity in regions flanking selected genes.
