# Supplementary material for: A Genomic Study of Myxomatous Mitral Valve Disease in Cavalier King Charles Spaniels
Source: Animals (Basel). 2020 Oct 16;10(10):1895. doi: 10.3390/ani10101895 (PMC7602727; doi:10.3390/ani10101895)
Supplement: Supplementary file 1 [file animals-10-01895-s001.pdf]

animals

Supplementary Materials: A Genomic Study of Myxomatous Mitral Valve Disease in Cavalier King Charles Spaniels

Arianna Bionda [1,2,†], Matteo Cortellari [1,2,†], Mara Bagardi [1], Stefano Frattini [1], Alessio Negro [1,2], Chiara Locatelli [1,*], Paola Giuseppina Brambilla [1] and Paola Crepaldi [1,2]

1 Department of Veterinary Medicine, University of Milan, Via dell'Università 6, 26900 Lodi, Italy; arianna.bionda@studenti.unimi.it (A.B.); matteo.cortellari@unimi.it (M.C.); mara.bagardi@unimi.it (M.B.); stefano.frattini@unimi.it (S.F.); alessio.negro@unimi.it (A.N.); paola.brambilla@unimi.it (P.G.B.); paola.crepaldi@unimi.it (P.C.)
2 Department of Agricultural and Environmental Sciences, University of Milan, Via Celoria 2, 20133 Milan, Italy
* Correspondence: chiara.locatelli@unimi.it; Tel.: +39-3398390588
† Co-first authors.

Received: 02 September 2020; Accepted: 12 October 2020; Published: date

Table S1. Proportion of cases and controls showing a run of homozygosity in regions flanking selected genes.

Gene	Cases (n = 16)	Controls (n = 17)	Difference between cases and controls
STEAP2	*0.55*	*0.09*	*0.46*
HEPACAM2	*0.50*	*0.05*	*0.45*
CDK6	*0.50*	*0.06*	*0.44*
ARNT2	*0.80*	*0.42*	*0.38*
KIAA1024	*0.79*	*0.41*	*0.38*
FAH	*0.80*	*0.42*	*0.38*
BCAR1	*0.44*	*0.10*	*0.34*
RAB10	*0.38*	*0.12*	*0.26*
PPP2R2C	*0.65*	*0.40*	*0.25*
LATS1	*0.57*	*0.34*	*0.23*
PDE1A	*0.62*	*0.43*	*0.19*
NRG1	*0.50*	*0.32*	*0.18*
TBC1D14	*0.59*	*0.44*	*0.15*
RAB3GAP1	*0.08*	*0.05*	*0.03*
UBXN4	*0.04*	*0.01*	*0.02*
ZRANB3	*0.04*	*0.02*	*0.01*
PLCB2	*0.19*	*0.17*	*0.01*
PDE3A	*0.32*	*0.32*	*0.00*
ADCY9	*0.00*	*0.00*	*0.00*
AXIN1	*0.00*	*0.00*	*0.00*
CACNA1H	*0.00*	*0.00*	*0.00*
CREBBP	*0.00*	*0.00*	*0.00*
PDPK1	*0.00*	*0.00*	*0.00*
SLC8A2	*0.00*	*0.00*	*0.00*
TRAP1	*0.00*	*0.00*	*0.00*
TCF7L1	*0.26*	*0.27*	*-0.01*
SMAD3	*0.20*	*0.24*	*-0.04*
TLN2	*0.30*	*0.35*	*-0.05*
WNT2	*0.08*	*0.18*	*-0.10*
CTNNAL1	*0.18*	*0.27*	*-0.10*
ADCYAP1R1	*0.06*	*0.18*	*-0.11*
FRRS1L	*0.17*	*0.28*	*-0.12*
EPB41L4B	*0.17*	*0.29*	*-0.12*
LPAR1	*0.17*	*0.29*	*-0.12*
CTNNA3	*0.06*	*0.20*	*-0.14*
PRKD1	*0.22*	*0.38*	*-0.17*
ADCY2	*0.25*	*0.43*	*-0.18*
ITPR2	*0.35*	*0.57*	*-0.22*
